# Dexamethasone suppresses the proliferation and migration of VSMCs by FAK in high glucose conditions

**DOI:** 10.1186/s40360-022-00604-3

**Published:** 2022-08-17

**Authors:** Ali Akbar Soleimani, Asghar Mohammadi, Ghasem Ghasempour, Borhan Rahimi Abkenar, Nafiseh Shokri, Mohammad Najafi

**Affiliations:** 1 Clinical Biochemistry Department, Faculty of Medicine, Iran University of Medical Sciences, Tehran, Iran; 2 Clinical Biochemistry Department, Faculty of Medicine, Tarbiat Modares University, Tehran, Iran; 3 Microbial Biotechnology Research Center, Iran University of Medical Sciences, Tehran, Iran

**Keywords:** VSMC, FAK, High-glucose, Migration, Dexamethasone

## Abstract

**Background:**

High glucose conditions cause some changes in the vessels of diabetes through the signal transduction pathways. Dexamethasone and other corticosteroids have a wide range of biological effects in immunological events. In the present study, the effects of dexamethasone were investigated on the VSMC (vascular smooth muscle cell) proliferation, and migration based on the FAK gene and protein changes in high glucose conditions.

**Methods and materials:**

The vascular smooth muscle cells were cultured in DMEM and were treated with dexamethasone (10^–7^ M, 10^–6^ M, and 10^–5^ M) for 24, and 48 h in high glucose conditions. The cell viability was estimated by MTT method. The FAK gene expression levels and pFAK protein values were determined by RT-qPCR and western blotting techniques, respectively. A scratch assay was used to evaluate cellular migration.

**Results:**

The FAK gene expression levels decreased significantly dependent on dexamethasone doses at 24 and 48 h. The pFAK protein values decreased significantly with a time lag at 24- and 48-h periods as compared with gene expression levels.

**Conclusion:**

The results showed that the inhibition of VSMC proliferation and migration by dexamethasone in the high glucose conditions may be related to the changes of FAK.

**Supplementary Information:**

The online version contains supplementary material available at 10.1186/s40360-022-00604-3.

## Introduction

 It is well known that biochemical and immunological factors, such as plasma lipoproteins, cytokines and some cellular changes such as endothelial and vascular smoot muscle cell dysfunctions may contribute to the development of atherosclerosis in diabetic individuals [1]. Moreover, high glucose conditions cause some structural and functional changes in the vessels of diabetes [2]. These changes develop fatty fibrous plaques (atheroma) and progress the stenosis and other complications in blood vessels [3, 4]. The vascular smooth muscle cells (VSMCs) are the most important vascular cells involved in the remodeling of these plaques [5, 6]. It is reported that the FAM3B is able to increase the VSMC proliferation and migration in the response to high glucose conditions [7]. Another study showed that anti-apoptotic proteins like Bcl-2, Bcl-xL, and Bfl-1/A1 upregulate and suppress VSMC apoptosis in high glucose conditions [8, 9]. Some proteins such as FAK (Focal Adhesion Kinase) are also reported to involve in the VSMC proliferation and migration events. It is phosphorylated through some integrin- and growth factor-related signaling pathways [10, 11].

Furthermore, dexamethasone and other corticosteroids have a wide range of biological effects in immunological events, diabetic macular edema and choroidal thickness [12–14]. Also, the most widely prescribed glucocorticoid for atherosclerosis treatment is dexamethasone [15, 16]. According to a study, dexamethasone’s inhibitory effects on platelet growth factor-induced VSMC migration are also mediated through PGC-1a regulation [17].

**Fig. 1:**
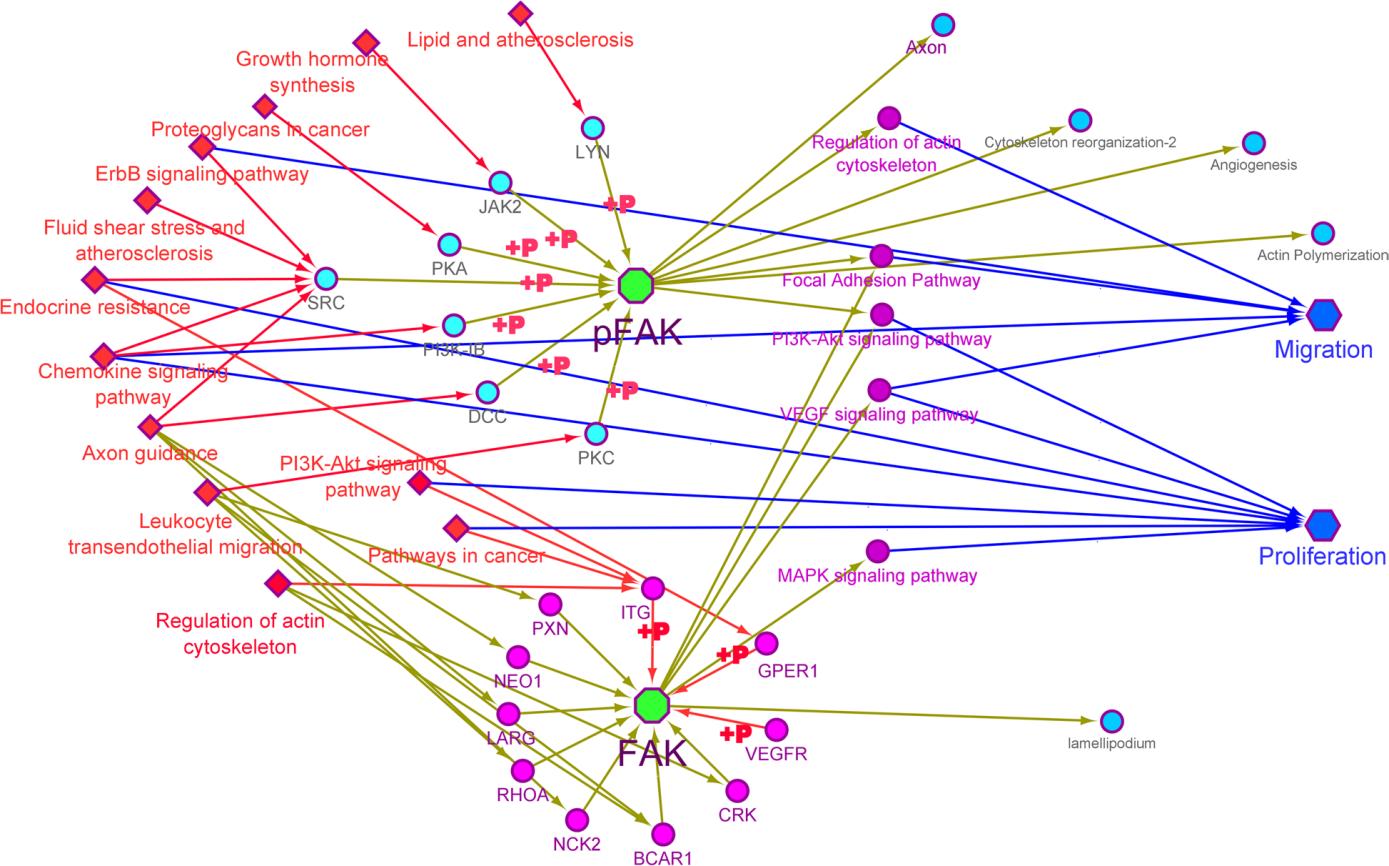
The roles of FAK and pFAK in signaling pathways. The list of signaling pathways were prepared through searching in KEGG server. Then, the FAK-related genes were enriched to the infection-unrelated metabolic pathways using Cytoscape software (https://cytoscape.org/). The FAK is phosphorylated by SRC, LYN, JAK2, PKA, DCC, PI3K and PCK genes or is auto-phosphorylated after interacting with ITG, VEGF and GPER1 receptors so that only pFAK transduces the cellular proliferation and migration events via cross talking with other signaling pathways or involving other downstream genes

In the present study, we went through searching the migration and proliferation keywords in KEGG (https://www.genome.jp/kegg) so that we considered the FAK as key gene in the infection-unrelated metabolic pathways. However, the non-phosphorylated FAK was able to interact with some proteins but the signal transduction was followed through cross-talking with other signaling pathways based on its phosphorylation by upstream enzymes or its autophosphorylation after interaction with membrane receptors (Fig. 1). Since the VSMC migration and proliferation events are mainly related to atherosclerosis process thus in this study, the effects of dexamethasone were investigated on the FAK gene expression levels and pFAK values, proliferation, and migration of vascular smooth muscle cells in high glucose conditions.

## Material & methods

### Cell culture

The vascular smooth muscle cells (NCBI Code, C591) were purchased from Pasteur Institute (Tehran, Iran). The cells were cultured in Dulbecco’s modified eagle medium (DMEM, Cat. No. BI 1004, Bioidea Company, Iran) with 10% Fetal Bovine Serum (FBS, Cat. No. 11560506, Gibco, USA) and 1% penicillin–streptomycin (Cat. No. BI1203, Bioidea Company, Iran). Then, the cell groups (70% confluency) including control normal, control high glucose (25 mM) [5], and dexamethasone (10^–7^ M, 10^–6^ M, and 10^–5^ M) plus high glucose (25 mM) were treated for 24, and 48 h.

### Cell viability

The MTT (Methyl Thiazol Tetrazolium, Sigma-Aldrich, USA) method was used to estimate the VSMC viability. In a 96-well plate, 4000 cells per well were seeded and cultured with different doses of dexamethasone for 24, and 48 h. The medium was then removed, and the seeded cells were incubated for 4 h at 37ºC with MTT solution (Cat. No. M5655, 0.5 mg/ml, Sigma-Aldrich, USA). After harvesting the MTT solution, 200 l$\upmu$ of DMSO (Dimethyl sulfoxide) was added to dissolve the produced crystals, and the mixture was shaken at room temperature for 15 min. Finally, optical density was determined at a wavelength of 570 nm.

### FAK gene expression

The GeneAll-Hybrid-R RNA purification kit (Cat. No. 305–101, Seoul, South Korea) was used to extract the total RNA from VSMCs. cDNA synthesis was carried out in accordance with producer’s instructions of SMOBIO kit (Hsinchu, Taiwan). For doing the real-time RT-PCR reaction, Amplicon Denmark’s SYBR Green PCR Master Mix (Cat. No. A190303, Amplicon Denmark) was used. The GAPDH gene has been used to normalize gene expression levels. The reactions for all genes were performed out in 15 µl volumes. Temperature cycles (45 cycles) were carried out at 95ºC (10 s) and 60ºC (45 s). To design gene primers, Primer-BLAST (NCBI.nlm.nih.gov/tools/primer-blast) and OligoCalc servers (http://biotools.nubic.northwestern.edu/OligoCalc.html) were utilized. The sequences of primers included for FAK gene, Forward; 5’- CATGCCCTCAACCAGGGATT -3’, Reverse; 5’- CACGCTGTCCGAAGTACAGT -3’ and for GAPDH, Forward; 5’-CATGAGAAGTATGACAACAGCCT-3’, Reverse; 5’-AGTCCTTCCACGATACCAAAGT-3’.

### Western blotting

To extract the total protein, RIPA buffer (Cat. No. sc-24948, Santa Cruz Biotechnology, USA) containing protease inhibitors (protease inhibitor cocktail and phenylmethylsulfonyl fluoride (PMSF)) was added to the cell plate and then, the shacked cell mixture was centrifuged at 13,000 g (4 °C, 20 min). The total protein value was determined using the Lowry method (Cat. No. TP0200-1KT, Sigma, USA). For 45 min at 90 V, 15 µl of protein sample was electrophoresed on a sodium dodecyl sulfate–polyacrylamide gel (12%). The protein bands were then transferred to a PVDF (polyvinylidene difluoride) membrane (Cat. No. IPVH00010, Merck Millipore, Darmstadt, Germany) for 60 min (90 V). The PVDF membrane was blocked with 4% fat-free milk (Nonfat Dry Milk, Cell Signaling Technology, USA) for 60 min before being incubated with a primary FAK antibody (1:1000 v/v, Cell Signaling Technology, Beverly, MA, USA, Cat. No. 3283 s) overnight at 4 °C. The PVDF membrane was then washed in tris-buffered saline containing 0.1 percent tween ® 20 (TBST) and was incubated with a secondary antibody (1:10,000 v/v, Cell Signaling Technology, Beverly, MA, USA, Cat. No. 7074 s) for 60 min at room temperature. Finally, the membrane was subjected to enhanced chemiluminescence (ECL) reagent (Cat. No. RPN2235, Amersham Biosciences, Italy). The protein values were normalized using beta-actin (1:1000 v/v, Cell Signaling Technology, Beverly, MA, USA, Cat. No. 4967 s). Image-J was used to identify the band densities.

### Scratch assay

A scratch assay was used to evaluate cellular migration. The VSMCs were cultivated in 12-well plates before being scraped with a pipette tip. After being washed with PBS (Cat. No. BCBS2233V, Sigma-Aldrich, Germany), the cells were cultured in the medium containing dexamethasone (10^–7^, 10^–6^, and 10^–5^ M) for 24, and 48 h. Images of the injured area were obtained using an inverted microscope (OPTIKA, Italy). Image J software was used to perform image analysis.

### Statistical analysis

Graphpad Prism (Version 8.0.3) was used to analyze the data. Initially, the Kolmogorov-Simonov test was used to assess data distribution. The results were then compared using the ANOVA test between the cell groups. The 2^−ΔΔCT^ formula was used to identify changes in gene expression. *p* < 0.05 was the subject to consider significant.

## Results

### Dexamethasone reduced the viability of VSMCs

**Fig. 2:**
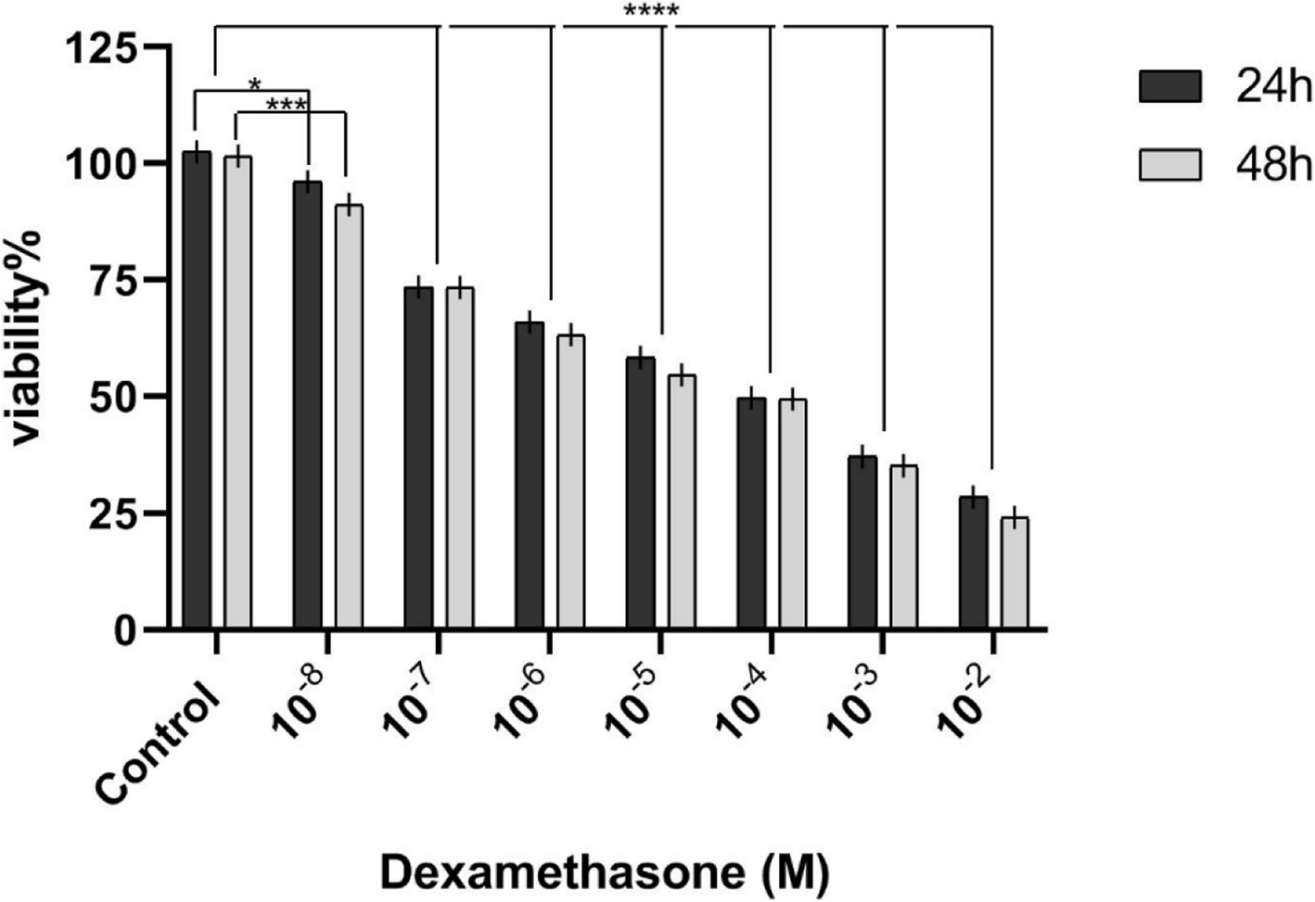
The effects of dexamethasone on the viability of VSMCs. The cellular viability was investigated for 24-h and 48-h periods. The cellular experiments were repeated three times (*n* = 3). Data are presented in mean and standard deviation (mean ± SD). * *p* < 0.05, ***p < 0.001, ****p < 0.0001

After being treated with various values of dexamethasone, the cell survival of vascular smooth muscle cells was measured. The treatment of VSMCs with dexamethasone significantly reduced cell survival and proliferation in a dose-dependent manner as compared to the control group after 24 and 48 h (Fig. 2).

### Dexamethasone reduced gene expression levels of FAK

**Fig. 3:**
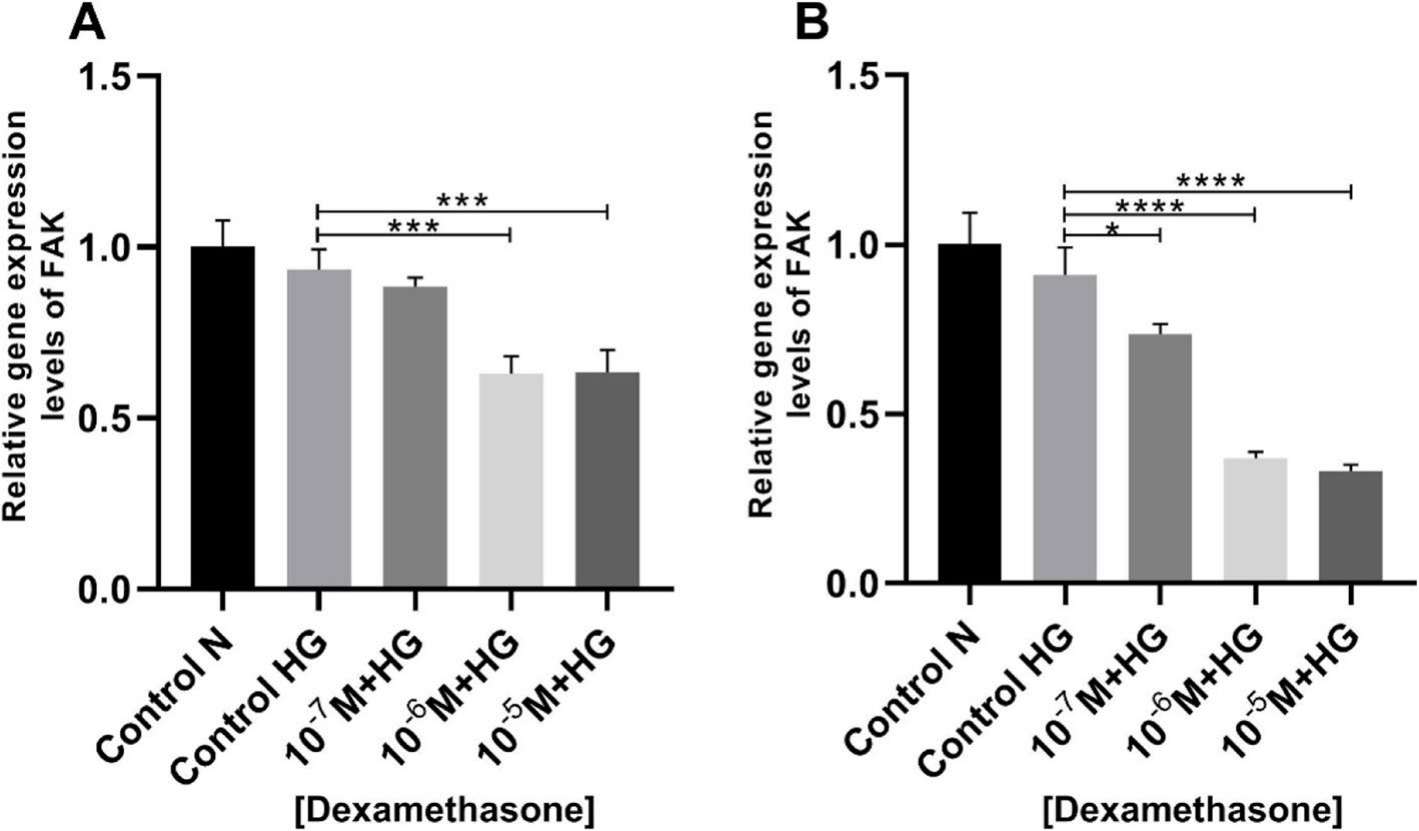
Focal adhesion kinase (FAK) gene expression levels in vascular smooth muscle cells (VSMCs) treated with dexamethasone. The FAK gene expression levels decreased significantly in both groups 24-h period **A** (control high glucose vs. dexamethasone (10^–7^ M + HG) *p* = 0.823; control high glucose vs. dexamethasone (10^–6^ M + HG) *P* = 0.0006; control high glucose vs. dexamethasone (10^–5^ M + HG) *p* = 0.0006), and 48-h period **B** (control high glucose vs. dexamethasone (10^–7^ M + HG) *p* = 0.0253; control high glucose vs. dexamethasone (10^–6^ M + HG) *p* < 0.0001; control high glucose vs. dexamethasone (10^–5^ M + HG) *p* < 0.0001). The cellular experiments were repeated three times (*n* = 3). Data are presented in mean and standard deviation (mean ± SD). * *p* < 0.05, ***p < 0.001, ****p < 0.0001. (N (Normal); HG (High Glucose))

The current investigation found that dexamethasone reduces the expression of the FAK gene in vascular smooth muscle cells after 24 and 48 h of treatment as compared to the control high glucose group (Fig. 3).

### Dexamethasone reduced pFAK protein values

**Fig. 4:**
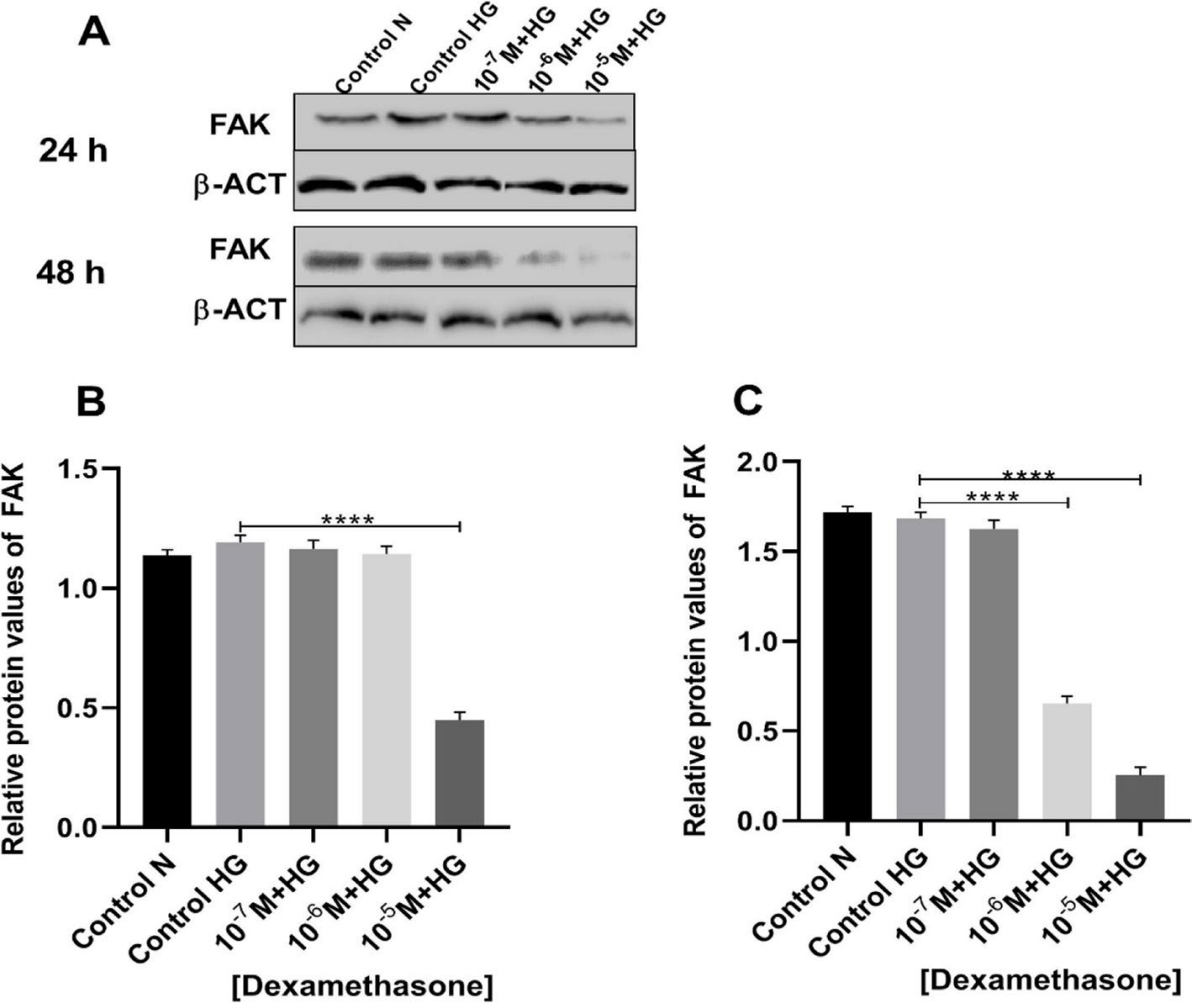
Relative protein values of FAK in vascular smooth muscle cells (VSMCs) treated with dexamethasone. Gel image **A** 24-h period **B** (control high glucose vs. dexamethasone (10^–7^ M + HG) *p* = 0.745; control high glucose vs. dexamethasone (10^–6^ M + HG) *P* = 0.307; control high glucose vs. dexamethasone (10^–5^ M + HG) *p* < 0.0001). 48-h period **C** (control high glucose vs. dexamethasone (10^–7^ M + HG) *p* = 0.430; control high glucose vs. dexamethasone (10^–6^ M + HG) *P* < 0.0001; control high glucose vs. dexamethasone (10^–5^ M + HG) *p* < 0.0001). The cellular experiments are repeated three times (*n* = 3). Data are presented in mean and standard deviation (mean ± SD). *** *p* < 0.001, **** *p* < 0.0001. (N (Normal); HG (High Glucose))

The pFAK protein values found to be significantly decreased in concentration of 10^–5^ M dexamethasone after 24 h (Fig. 4 A and B). On the other hand, a significant decrease in pFAK protein value was observed after 48 h of treatment with dexamethasone in values of 10^–6^ and 10^–5^ M (Fig. 4 A and C).

### Dexamethasone reduced the cell migration of VSMCs

**Fig. 5:**
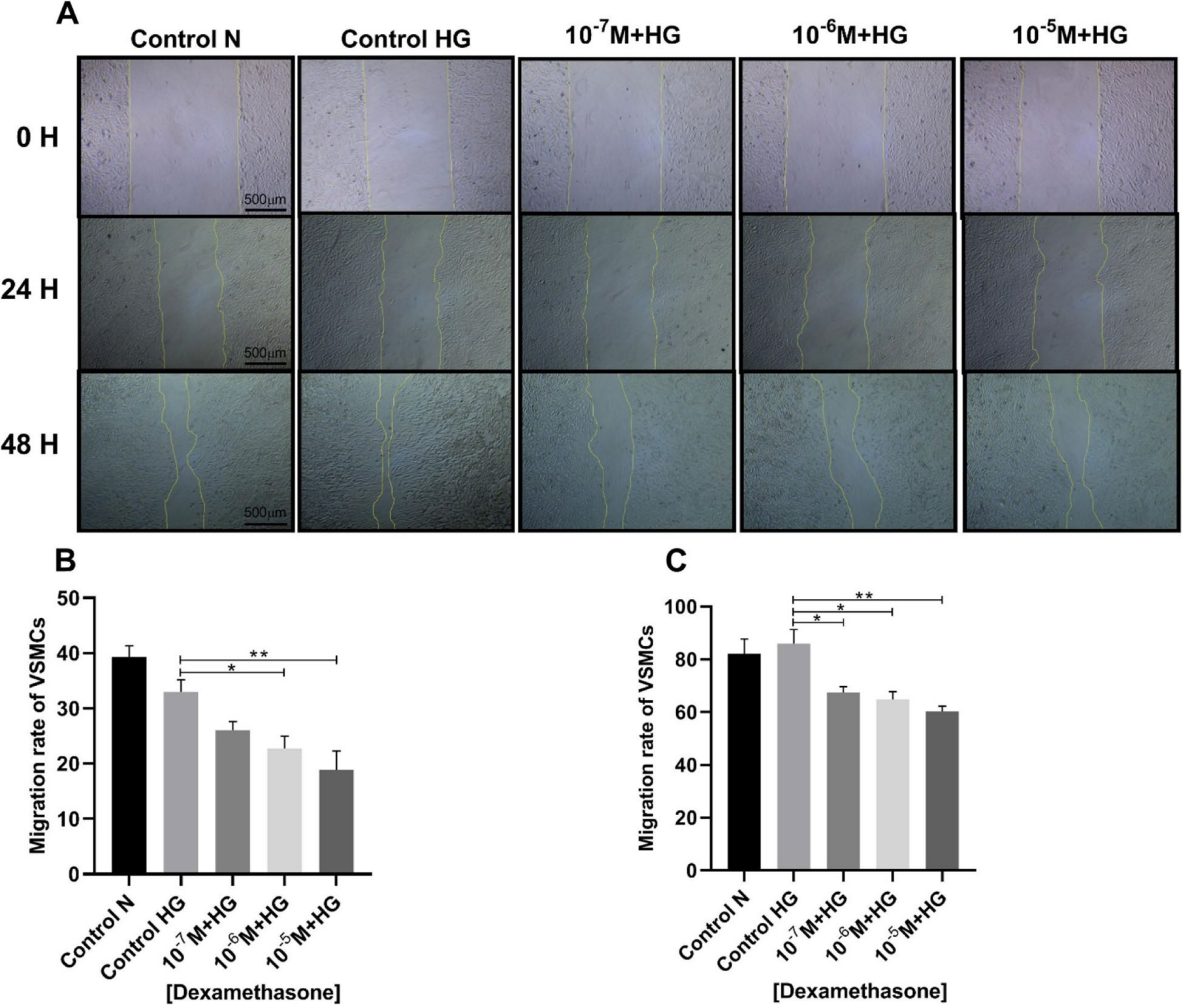
The effects of dexamethasone on the migration of VSMCs. **A** Microscopic images of VSMCs cultured with dexamethasone. 24-h period **B** (control high glucose vs. dexamethasone (10^–7^ M + HG) *p* = 0.144; control high glucose vs. dexamethasone (10^–6^ M + HG) *p* = 0.0273; control high glucose vs. dexamethasone (10^–5^ M + HG) *p* = 0.0099). 48-h period **C** (control high glucose vs. dexamethasone (10^–7^ M + HG) *p* = 0.0276; control high glucose vs. dexamethasone (10^–6^ M + HG) *p* = 0.0158; control high glucose vs. dexamethasone (10^–5^ M + HG) *p* = 0.0067). * *p* < 0.05, ** *p* < 0.01. (N (Normal); HG (High Glucose))

The study of the migration rate of vascular smooth muscle cells showed that the dexamethasone (10^–6^ and 10^–5^ M) significantly decreased the migration in period 24 h (Fig. 5 A and B). Additionally, a significant decrease in cell migration was observed in all three concentrations of dexamethasone (10^–7^, 10^–6^ and 10^–5^ M) after 48 h (Fig. 5 A and C).

## Discussion

According to several studies, hyperglycemia is a paraclinical feature of diabetes that has been linked to VSMCs proliferation and migration in atherosclerosis. The high glucose conditions promote the VSMC proliferation, which is mediated in part by HG-activated STAT3/Pim-1 signaling [18]. Another study found that FAM3B regulates VSMC proliferation and migration in response to high glucose induced by inhibiting miR-322-5p [7]. Atherosclerosis is a well-known inflammatory disease of the vascular wall that is recognized as the underlying cause of CVDs, including hypertension, myocardial infarction, stroke, and peripheral arterial disease [19]. Many agents such as inflammation, angiogenesis, hypertension, genetics, diabetes, and life style are related to the atherogenesis process [2, 20]. It is generally understood that VSMC proliferation and migration are critical biological factors in atherosclerosis progression [21, 22]. Previous studies showed that VSMCs play a multifaceted role in atherosclerotic lesions, and that depending on the context, they can improve plaque stability and rupture [23]. Also, the studies revealed that growth factors and cytokines stimulate the proliferation and migration of VSMCs in the vessel wall’s intermediate layer [24, 25]. As a result, more understanding of how VSMCs function in plaque is required to develop effective treatment strategies to reduce cardiovascular risk.

It is well known that several signaling pathways related to cellular proliferation and motility are involved through the core protein of FAK [11, 26]. The FAK-mediated pathways are activated by phosphorylation and auto-phosphorylation events so that the pFAK transduces the signals to the more internal parts of cells. The regulation of cell migration by integrin-FAK pathway, in particular, is well recognized in many cell types that relates to the progression of cancer and other diseases [27, 28]. It is well known that FAK, as an allosteric enzyme, must be phosphorylated (pFAK) to transfer the messages downstream of pathways. Based on the cell interventional studies, there were no significant correlations between the FAK activity and total protein [29]. Moreover, there was a decrease in the total protein pool because of the necrotic roles of dexamethasone. Thus, the active form of FAK (pFAK) was studied as the most important factor triggering biological pathways in this study, however, the total FAK may give more data on the enzyme active/inactive ratios.

Dexamethasone has multiple biological effects, particularly in the regulation and suppression of immunological function. Some studies have also shown that dexamethasone can influence the migration of vascular smooth muscle cells and the development of atherosclerosis by several molecular pathways [12, 17, 30]. These studies reported that the proliferation and migration of vascular smooth muscle cells are suppressed by up-regulating PGC-1α expression [17] and modulating matrix metalloproteinase activity [30]. In the agreement with these reports, this study showed that the VSMCs treated with dexamethasone in the values of higher than 60% of cellular viability inhibit both the cellular proliferation and migration. Furthermore, the results showed that the FAK gene expression and phosphorylated protein levels decrease in the dexamethasone-treated VSMCs. Sine the FAK is a central protein in many biological pathways that mediate the cellular migration and proliferation processes thus we resulted that dexamethasone addition to the signaling pathways suggested in other studies may be suppress the FAK-induced signaling pathways in the VSMCs.

## Conclusion

The results showed that the cellular proliferation and migration are inhibited in the VSMCs treated with dexamethasone. Also, the FAK gene expression and phosphorylated protein levels decreased so that we suggested that the pFAK-induced signaling pathways may be suppressed in the dexamethasone-treated VSMCs. More studies should investigate the phenotype changes of cellular scaffolds.

## Supplementary Information

**Additional file 1.**

**Additional file 2.**

**Additional file 3.**

## Data Availability

The datasets used and/or analyzed during the current study are included as supplemental materials.

## Abbreviations

FAK: Focal Adhesion Kinase
VSMCs: Vascular Smooth Muscle Cells
CVD: Cardiovascular diseases
Dexa: Dexamethasone
HG: High glucose
DMEM: Dulbecco’s Modified Eagle Medium
FBS: Fetal Bovine Serum
PBS: Phosphate-Buffered Saline
cDNA: Complementary DNA
MTT: Methylthiazol tetrazolium
PMSF: Phenylmethylsulfonyl Fluoride
PVDF: Polyvinylidene Difluoride
TBST: Tris-Buffered Saline with 0.1% Tween ® 20
ECL: Enhanced Chemiluminescence
EGFR: Epidermal growth factor receptor
