# Supplementary material for: Dexamethasone suppresses the proliferation and migration of VSMCs by FAK in high glucose conditions
Source: BMC Pharmacol Toxicol. 2022 Aug 17;23:63. doi: 10.1186/s40360-022-00604-3 (PMC9382766; doi:10.1186/s40360-022-00604-3)
Supplement: Supplementary file 1 — Additional file 1. [file 40360_2022_604_MOESM1_ESM.pdf]

(A) 24-hour Period

FAK

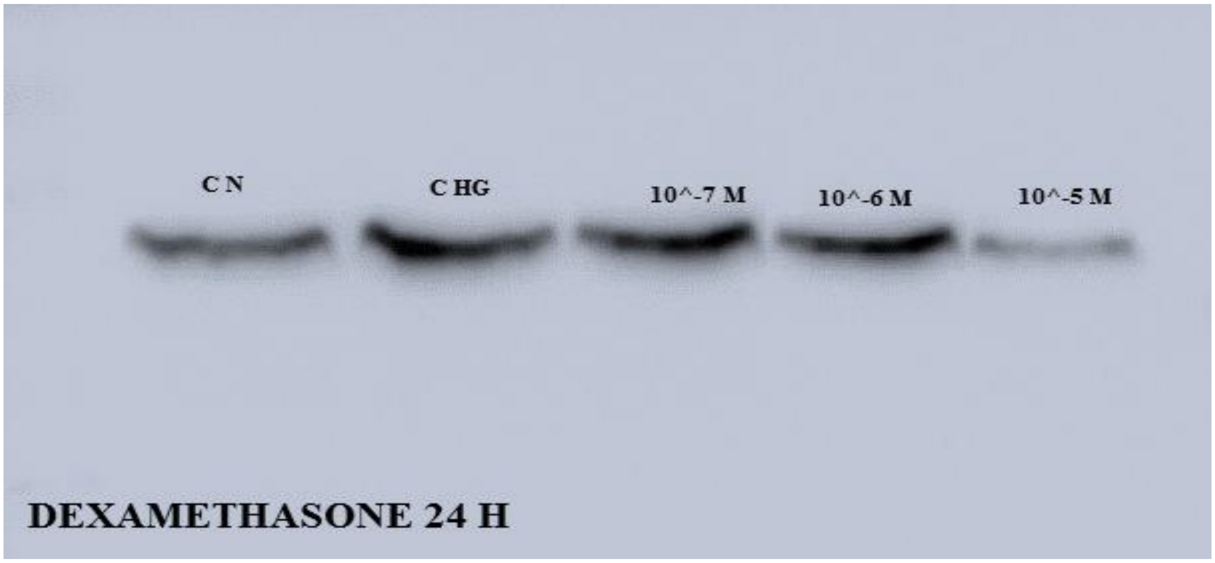

CN = Control normal, C HG= Control high glucose

B-ACTIN

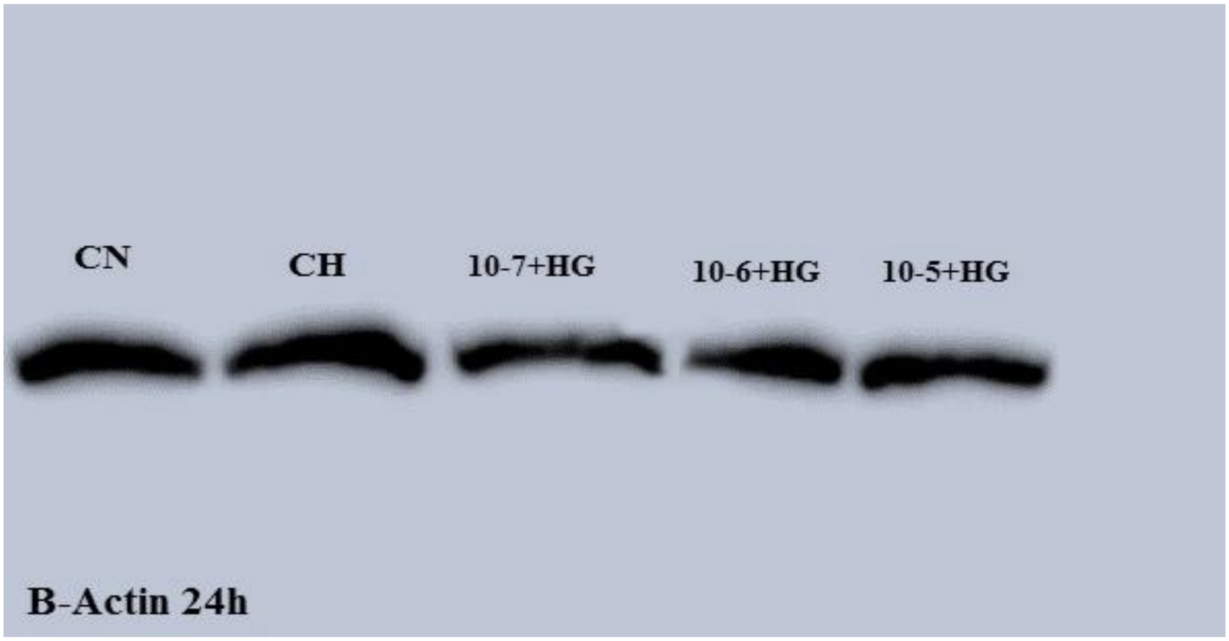

CN = Control normal, CH= Control high glucose

(B) 48-hour Period

FAK

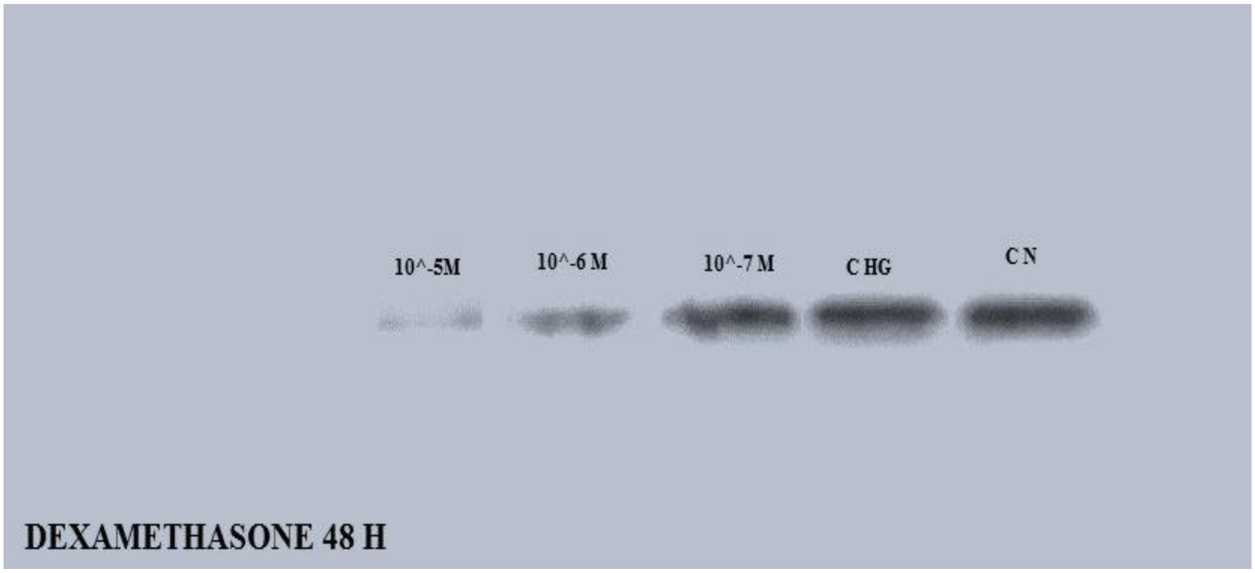

CN = Control normal, C HG= Control high glucose

B-ACTIN

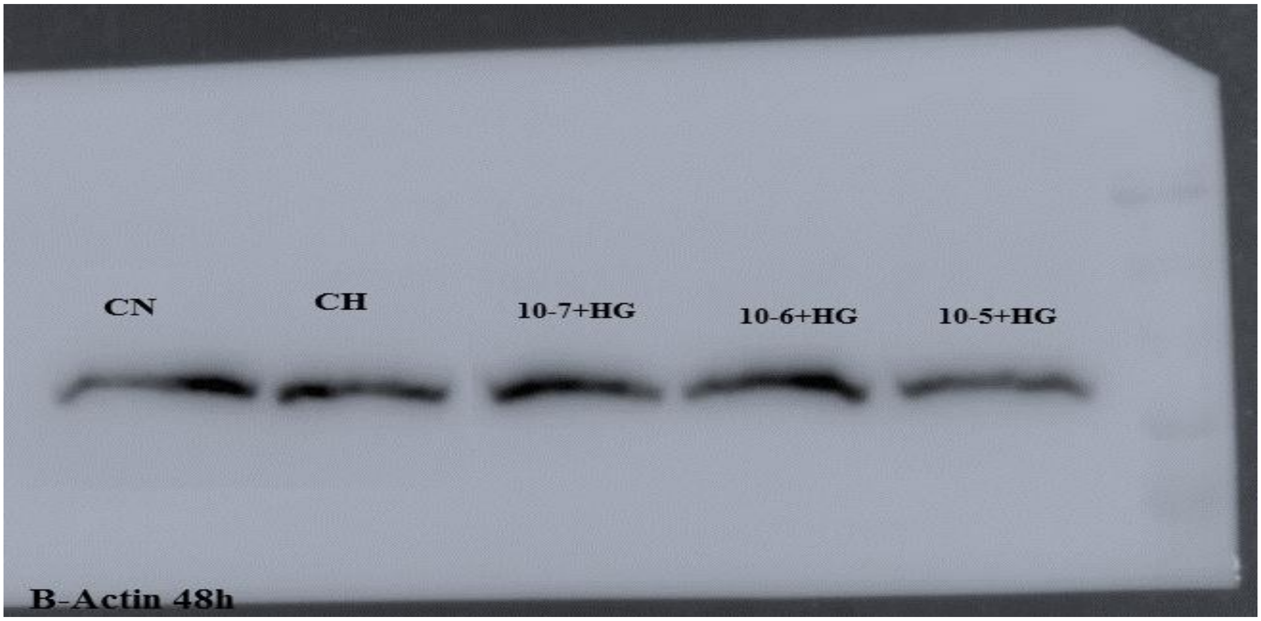

CN = Control normal, CH= Control high glucose

Supplement 1: The discontinuous SDS-PAGE technique was used for protein separation. The cellular lysates were loaded and run on the stacking gel (%5). After the separation of proteins on the separating gel (%12), the stacking gel was removed so the separating gel was the subject for transferring of the proteins on PVDF membrane, its incubation with the primary and secondary antibodies, and the enhancement of band chemiluminescence. Then, the images obtained from the PVDF membranes were cropped to show better protein bands in the manuscript.
