# Supplementary material for: Dexamethasone suppresses the proliferation and migration of VSMCs by FAK in high glucose conditions
Source: BMC Pharmacol Toxicol. 2022 Aug 17;23:63. doi: 10.1186/s40360-022-00604-3 (PMC9382766; doi:10.1186/s40360-022-00604-3)

VSMC Migration

(A): Zero-hour (0H) group

Control normal

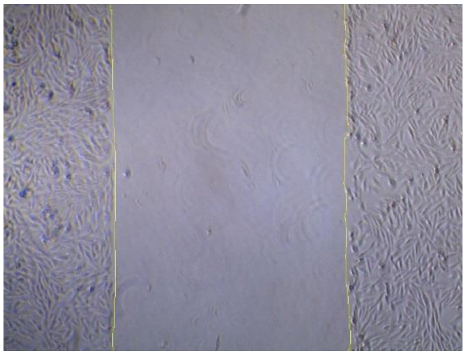

Control high glucose

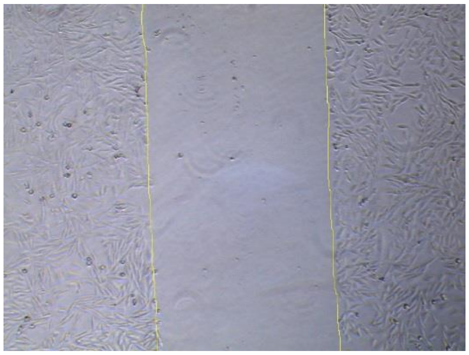

Dexamethasone 10^{-7} M

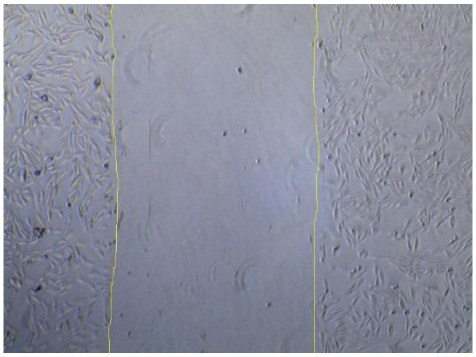

Dexamethasone 10^{-6} M

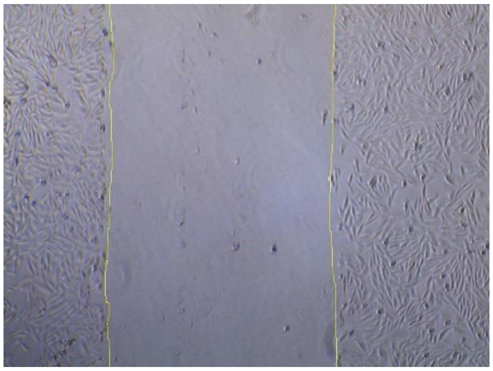

Dexamethasone 10^{-5} M

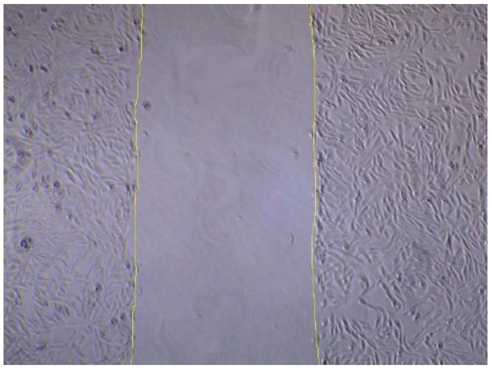

(B): 24-hour group

Control normal

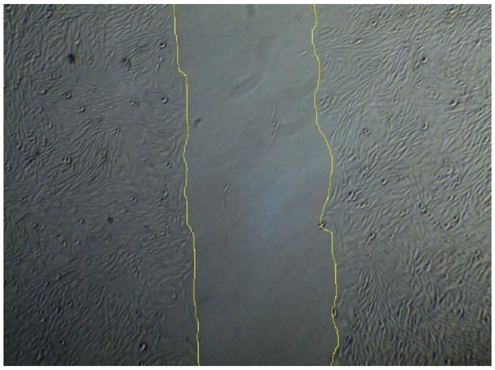

Control high glucose

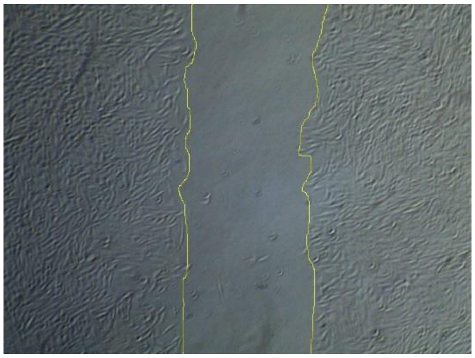

Dexamethasone 10^{-7} M

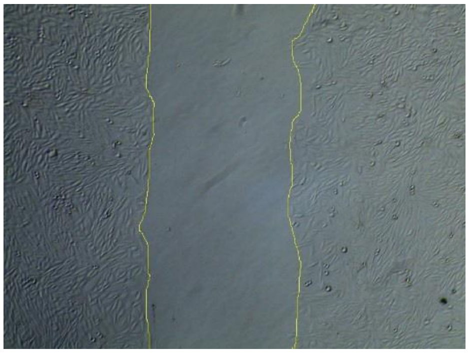

Dexamethasone 10^{-6} M

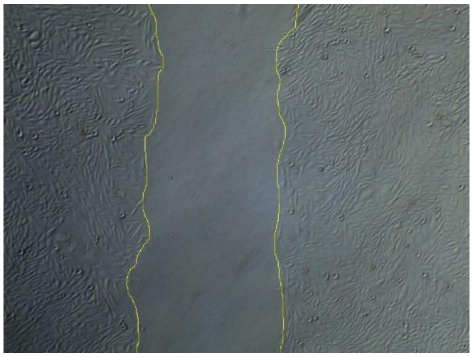

Dexamethasone 10^{-5} M

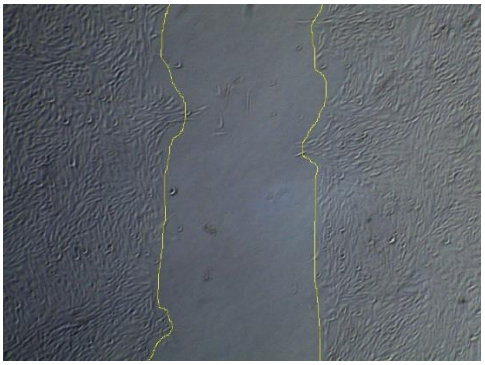

(C): 48-hour period

Control normal

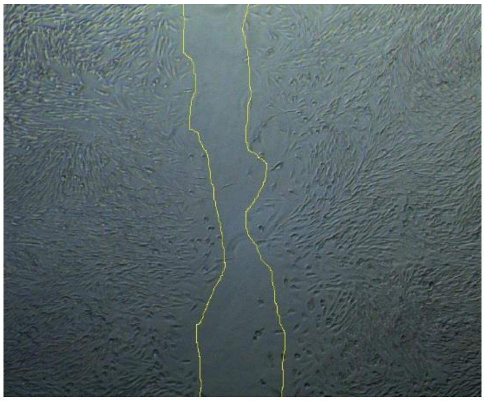

Control high glucose

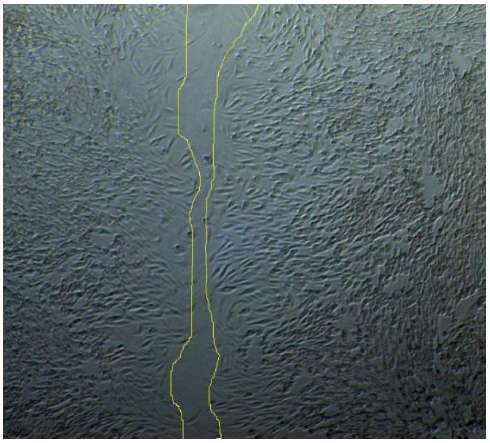

Dexamethasone 10^{-7} M

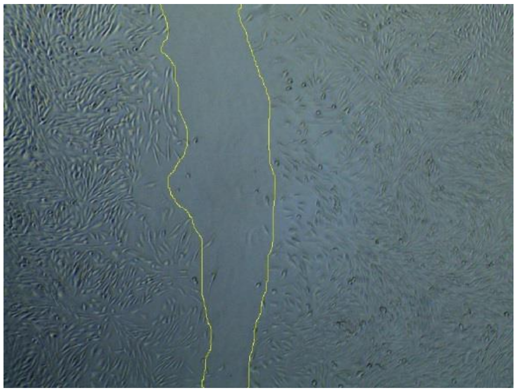

Dexamethasone 10^{-6} M

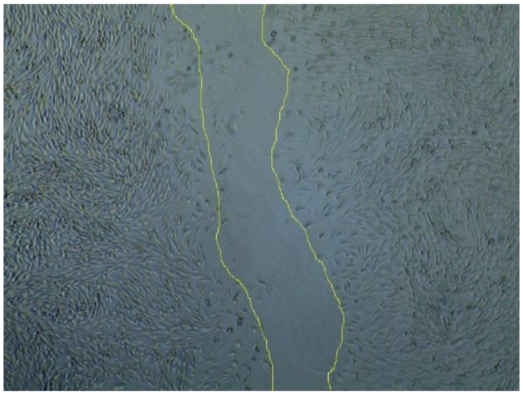

Dexamethasone 10^{-5} M

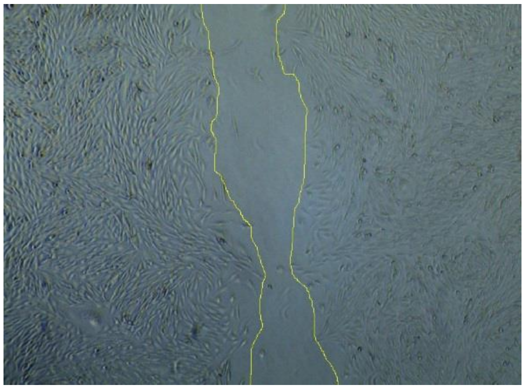

Supplement: Supplementary file 2 — Additional file 2. [file 40360_2022_604_MOESM2_ESM.pdf]
